# Effect of online solution-focused counseling on the sexual quality of life of women with a history of breast cancer: a clinical trial

**DOI:** 10.1186/s12905-023-02468-z

**Published:** 2023-06-21

**Authors:** Mahshid Bokaie, Nafiseh Sadat Hejazi, Mirsaeed Jafari, Masoud Shabani

**Affiliations:** 1 Research Center for Nursing and Midwifery Care, Shahid Sadoughi University of Medical Sciences, Yazd, Iran; 2 Student Research Committee, Shahid Sadoughi University of Medical Sciences, Yazd, Iran; 3 Refah University, Tehran, Iran; 4 Shahid Ramezanzadeh Radiation Oncology Center, Shahid Sadoughi University of Medical Sciences, Yazd, Iran

**Keywords:** Breast cancer, Counseling, Solution-focused, Sexual quality of life, Online counseling

## Abstract

**Background:**

Breast cancer, as the most prevalent malignancy among women globally and in Iran, affects important aspects of the lives of the survivors of this condition, especially the quality of sexual life. Solution-focused brief therapy is one of the therapeutic counseling approaches used in various fields, including sexual function problems. In recent years, online and internet-mediated counseling methods have received more attention.

**Objective:**

This study investigated the effect of online counseling based on a solution-focused approach in improving the sexual quality of life (SQL) of women with breast cancer.

**Materials & methods:**

In this clinical trial, the research population consisted of women with a history of breast cancer with a recorded file in Shahid Ramazanzadeh Radiation Oncology Center in Yazd and at least 6 months had elapsed since the end of their treatment. After selecting 80 samples based on the random numbers table, they were assigned into two groups of 40, i.e., active control and intervention, using random allocation software. Participants in the intervention group were given online counseling through the Skyroom space with a solution-focused approach. Eight weekly sessions were held each lasting 60–90 min. The active control group received an educational file to improve the SQL. All participants in two groups completed the SQL questionnaire at the beginning of the study, at the end of the study, and 1 month after the study. Data were analyzed with SPSS18 using descriptive and inferential statistics.

**Results:**

Finally, the data of 33 participants in the intervention group and 32 participants in the active control group were analyzed. The mean score of the SQL in the intervention group increased from 68.57 ± 18.63 before the intervention to 78.84 ± 12.7 immediately after the intervention (*P* = 0.015), and to 79.60 ± 19.88 1 month after the intervention (*P* = 0.012). The mean score of the SQL in the active control group increased from 64.45 ± 22.76 before the intervention to 67.20 ± 20.29 immediately after the intervention (*P* = 0.33), and to 68.70 ± 20.76 1 month after the intervention (*P* = 0.62). The difference in the mean score of the SQL before and after the intervention between the two groups was statistically significant (*P* = 0.007).

**Conclusion:**

It seems that the use of counseling with a solution-focused approach in women with a history of breast cancer improves the SQL. Thus, considering the effectiveness of this type of training, it is recommended that this method be used as a sexual health counseling method in medical centers.

**Trial registration:**

This clinical trial is registered in Iranian registry center of clinical trials (IRCT) by registration code of IRCT20201221049784N1 in 06/03/2021.

## Introduction

Breast cancer(BC) is rendered as the most prevalent malignancy in women, which accounts for 11.6% of all cancers worldwide [1]. It is the second leading cause of cancer-related death among all types of cancer in women [2]. According to the report by the World Health Organization (WHO), BC has a prevalence rate of 12.5% among all cancers and is the most common type of cancer among Iranian women [3]. Its annual incidence is more than 1.7 million new cases per year worldwide [4]. Recent studies suggest a growing increase in its annual incidence [5]. Affliction with breast cancer can be an important factor in creating numerous psychological consequences and sexual problems such as sexual dysfunction, disturbance in sexual performance (dyspareunia, fatigue, vaginal dryness, breast sensation disorder, and lack of sexual orgasm) and sexual intimacy due to diminished physical strength, reduced ability to perform daily routines, hospitalization, and finally the resulting depression [6]. Breast cancer treatment methods can also lead to the occurrence of many psychological consequences, including depression and anxiety for the patient. Breast, as an influential organ in a woman’s body image, has a significant role in the sexual aspects of a person’s life, and surgical procedures can cause a disturbance in the body image, femininity, and sexual quality of life by changing the shape or creating defect in this organ [7, 8]. Radiotherapy and chemotherapy have also caused a wide range of physical, psychological, and social symptoms in studies [9]. In a study conducted by Sbitti et al. in Morocco, the symptoms of sexual dysfunction including dyspareunia (65%), lubrication disorder (54%), lack of sexual satisfaction (48%), and orgasm disorder (40%) have been observed in women following breast cancer treatment. Ninety percent of cases of sexual dysfunction occurred after chemotherapy and 9% after surgery, and overall, it affected the sexual quality of life of these patients [10]. Hence, the quality of life of these women, consisting of their sexual function, is mainly influenced by the diagnosis and treatment of BC [11]. Sexual function is an essential aspect of patients’ well-being, because sexual issues can affect their quality of life and persist long after the end of treatment [12]. Many studies have shown that there is a direct relationship between sex and quality of life [13–15]. Sexual quality of life is a picture of the state of health and the general quality of life of participants [16] and is defined by factors such as the feeling of sexual attractiveness, interest and participation in sexual relations and the perception of sexual performance [17]. The sexual quality of life is one of the main concerns in women with breast cancer, so that according to studies, between 68–70% of these participants have experienced one of the manifestations of sexual dysfunction [18]. Given the increased survival rate of patients following the advances in diagnostic and therapeutic modalities, the possibility of patients’ involvement in the long-term effects of breast cancer in the quality of life increases and the need to foresee supportive measures and counseling treatments in the protocol is highlighted more than ever [19]. In the studies conducted on breast cancer survivors, a variety of counseling treatments with different approaches, such as exploring the effect of psychosexual treatment [20], use of the PLISSIT model [21], solution-focused group intervention [22], and cognitive-behavioral therapy [23] on the performance and sexual quality of life of women with breast cancer have been investigated. The solution-focused approach is one of the approaches that has received less attention in previous studies. This approach is a subset of Positive Psychology School [24], which was first established in 1980 by Berg & De Shazer [25]. They believed that trying to solve the problem often causes the continuation of the problem and that understanding the roots of the problem is not always necessary [26]. This approach was gradually expanded by different researchers in the last five decades, and its first treatment guideline was developed in 2008 by the SFBT association (Solution-Focused Brief Therapy) and was revised in 2013 [27]. In this approach, the problem has the second priority and paying attention to exceptional situations is the first priority [28]. In other approaches, the therapist focuses more on the client’s past; yet, in this approach, s/he looks at the present and the future, and the therapist’s effort is in the direction of how the client can manage her problems and distinguish the behaviors that cause the problem to continue [29]. Moreover, in this approach, the therapist tries to create a reciprocal cooperation with the client to give them a role in solving the problem. One of the most important advantages of the solution-focused approach is the limited number of sessions, which predisposes to better acceptance and reduced costs [30]. Counseling is used as a common method to improve the sexual quality of life, and can be done in two ways, face-to-face and online [31]. The occurrence of the Covid-19 pandemic at the end of 2019 and the restrictions imposed on more communities highlights the importance of using non-face-to-face counseling methods and the necessity of more study and research in this field. The high prevalence and increasing incidence of breast cancer in Iran, the prevalence of mental and psychological problems, and the decline in the sexual quality of life in women who survived this cancer, less attention to the solution-focused approach in improving the sexual quality of life in past studies, the advantages of this approach, and the new conditions governing human societies following the Covid-19 pandemic were the factors that led us to conduct this study with the aim of investigating the effect of online counseling based on a solution-focused approach in improving the sexual quality of life of women with breast cancer undergoing treatment.

## Methodology

### Design and setting

This interventional parallel clinical trial was conducted between February 2021 and December 2022 using before-after design with an active control group and a 1-month follow-up. The statistical population of this research was all women with breast cancer who had referred to Shahid Ramezanzadeh Radiation Oncology Center in Yazd for treatment. To conduct the research, written informed consent was obtained from all the participants. Eligible participants were contacted based on the list available in the center according to the inclusion criteria. The samples were selected based on the inclusion criteria by reviewing the available records. Among the participants who had referred to Ramazanzadeh Radiation Oncology Center in Yazd, the information of 550 patients with breast cancer was checked and of these, 165 participants were invited to participate in the study through phone calls. Finally, 80 participants who met the inclusion criteria entered the study. The required sample was determined using the following formula in each group.

$$n=\frac{{\left({Z}_{1-\alpha /2}+{Z}_{1-\beta }\right)}^{2}\left({S}_{1}^{2}+{S}_{2}^{2}\right)}{{\left({\mu }_{1}-{\mu }_{2}\right)}^{2}}$$

According to Hummel et al.’s paper [23] and taking into account the standard deviation of the quality score of the intervention and active control groups as 8 and 7.1, respectively, confidence level was 95%, test power of 80%, the minimum mean difference of 5.7, and subject attrition rate of 10%, 40 samples were considered in each group.

### Eligibility criteria

Inclusion criteria were: willingness to participate in the study, age between 18 and 45 years, being married and sexually active, a history of breast cancer, passage of at least 6 months since the end of treatment, no disease, no history of participating in sexual health education and counseling sessions, and access to the internet and a smart phone and couples do not have another permanent or temporary partner. Exclusion criteria were: affliction of the patient or her spouse with chronic diseases affecting sexual relations such as diabetes and cardiovascular disease, consumption of any drug affecting sexual relations by the patient or her spouse (self-reported), affliction with other types of cancer, and patient’s or her spouse’s affliction with serious psychological disorders such as severe depression and psychosis and severe conflict between couples.

The first-generation random allocation method was used to assign the eligible participants into two intervention and active control groups.

Each participant was given a number from 1 to 80.

Then, using the website http://www.randomization.com, participants were randomly assigned into two blocks of 40, A and B.

The selection of each block as intervention or active control group was done by a person outside the study and based on lottery.

### Instruments

The demographic information form and the Farsi version of women’s sexual quality of life questionnaire were both developed electronically and the link was provided to the participants (https://survey.porsline.ir/s/ze3Px6W, https://survey.porsline.ir/s/Jo0HLhy).

#### Demographic information form

This included patient’s age, spouse’s age, patient’s education, spouse’s education, patient’s employment status, spouse’s employment status, and information on breast cancer including: time of diagnosis, type of tumor, type of treatment, surgical treatment method, number of radiotherapy sessions, number of chemotherapy sessions, duration of recovery, and the history of cancer in the patient and his family.

#### Sexual quality of life questionnaire

This questionnaire was developed for the first time in 1998 and was revised and validated in 2005 by Symonds et al. It consists of 18 items with a 6-point Likert scale. The minimum score obtained from this tool is 18 and the maximum score is 108. A higher score indicates a better level of sexual quality of life. According to the range of answers to the items, a score of up to 36 is classified as a poor class, a score of 72-37 as an average class, and a score of 108-73 as a favorable class [32]. Besides, the validity and reliability of the Farsi version of this questionnaire was first confirmed by Ma’soumi et al. [33] with Cronbach’s α of 0.73 in Iran.

### Interventions

To conduct the research, informed consent was obtained from the participants by creating an online informed consent form and sending the relevant link. After obtaining consent and explaining the research objectives, demographic information forms and sexual quality of life questionnaires were also provided online to all participants. After completing the questionnaires, the participants of the intervention group were added to a group in WhatsApp application to receive information and guidance related to the intervention process and participation in counseling sessions. In the process of implementing the intervention sessions, to better interact and provide the possibility of questions and answers between the participants and the consultant, the participants of the intervention group were divided into two blocks of 20 and entered in two separate rooms in the Skyroom space. The sessions of each block were held at two separate times every other day. The intervention consisted of eight 90-min counseling sessions with a solution-focused approach, which were held online once a week. A survey was conducted among the participants in the WhatsApp group regarding the day and time of holding the sessions. The sessions were held online using the Skyroom space. In each session, the researcher presented solutions to solve the problems based on the solution-focused approach protocol using the common tools of the approach. At the end of each session, assignments were determined for the participants wherein they were required to present them at the beginning of the next session. The last 15 min of each session were devoted to questions and answers and clarifying the doubts of the participants. In the time between the sessions, the participants could raise their questions in the group and receive answers. If a person was absent from a session, the contents of that session would be provided to the person. Participants not taking part in more than one session or not doing the assignment in more than two sessions were rendered as refusing to continue cooperation (details of the content of the sessions, Table 1). During the intervention, out of a total of 80 participants who entered the study, 15 participants were excluded from the study due to the reasons of refusing to continue participating in the study, not actively participating in the sessions, and not completing the assignments; finally, 65 participants cooperated until the end of the study, including 33 participants in the intervention group (18 participants in the first block and 15 participants in the second block) and 32 participants in the control group. Each member of the active control group was also sent an educational pamphlet on sexual issues through WhatsApp application to read every 2 weeks. Immediately after the end of the intervention period and 1 month after its completion, the participants of the intervention and control groups completed the sexual quality of life questionnaire again.

**Table 1:** Details of the content presented in the sessions with a solution-focused approach

**Session**	**Session goal**	**Session content**	**Home practice**
First	Getting to know Statement of rules Vision	Getting to know the group members with each other Explaining the goals and vision of the group Stating the rules, norms and duties of the members.	Who should I ask? Determining the order of introduction of members by each other. A statement of preferred goals for each member
Second	Targeting	Review the assignment of the previous session Examining people’s highest hopes Focus on small changes.	Group exercise determining the highest hopes of the members. Examining the changes that people have experienced recently.
Third	Highlighting abilities	How did you improve your sexual performance after breast cancer?	Prepare a list of your abilities and skills from the beginning of the course
Fourth	Discovery Exceptions	Women are encouraged to review situations in which the current problem did not exist.	Visualizing a future without breast cancer
Fifth	Dominance and power	Generalize new solutions to other situations Focusing on the mastery and strength of each member	Focusing on application situations of new solutions Identify unused abilities and skills
Sixth	Feedback and admiration	Feedback and discussion about new experiences	Focusing on positive experiences and presenting positive self-talk
Seventh	A celebration of change	Review the benefits of using solutions Thanks for the changes	Appreciate each other for mutual influence and help. Write a loving letter to yourself.
Eighth	Conclusion	Reviewing the achievements and setting the follow-up meeting	Preparation of birth certificates of skills and achievements until the follow-up period.

### Ethical considerations

This study was conducted based on the Declaration of Helsinki and the approval of the Ethics Committee of the Research Vice-Chancellor of Shahid Sadoughi University of Medical Sciences with code: IR.SSU.REC.1399.260. Additionally, after explaining the objectives of the study to the participants, informed written consent was obtained from all the participants to take part in the study and they were assured of the confidentiality of the study results. This clinical trial has been registered in the Iranian Registry of Clinical Trials (IRCT) with the code: IRCT20201221049784N1 in 06/03/2021.

### Data analysis

Statistical analysis of extracted data was done using SPSS18 (SPSS, Inc., Chicago, IL, USA). Descriptive statistics were used to describe the data, draw tables, and calculate the percentage, mean and standard deviation. Inferential statistics were used to compare the mean of the variables in each group. Chi-square test was used to compare qualitative variables.

The one sample Kolmogorov-Smirnov test will be used to check the normality of the distribution of the study data. Due to the non-normality of the distribution of the quantitative variables related to the demographic form, the Mann-Whiteny non-parametric test was used to compare the variables in the two groups. Also, due to the non-normality of the data distribution, Wilcoxon test was used to compare the mean score of the sexual quality of life at three time points for each group. Further, due to the non-normality of data distribution, the Mann-Whitney test and the between-Subjects Effects test were used to compare the score of the sexual quality of life between the two groups at three points in time (*P* < 0.05).

## Results

**Fig. 1:**
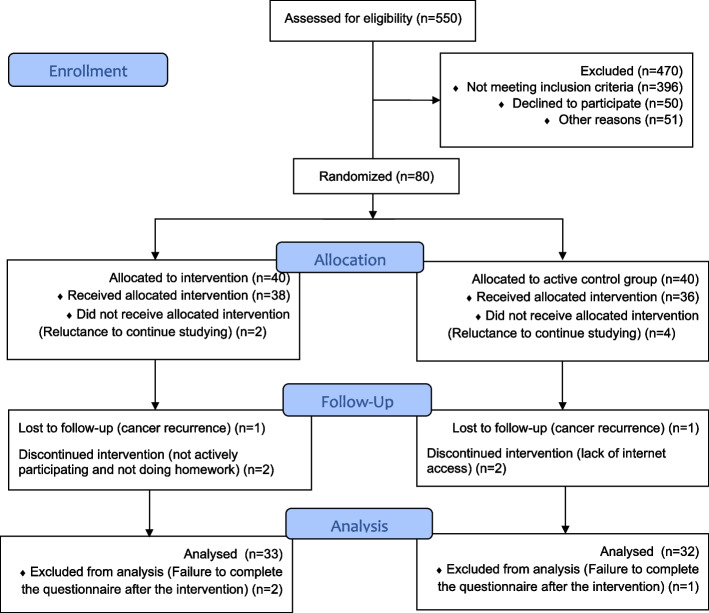
CONSORT Flow Diagram

Out of 80 people who entered the study based on eligibility criteria, 15 people were excluded from the study for various reasons, and finally the data of 65 people (32 people in the intervention group and 33 people in the active control group) were statistically analyzed. For each group, the losses and exclusions after randomization with reasons are shown in detail in Fig. 1.

The results showed that the mean age of participants was 40.6 ± 4.05 years. Also, 62.8% of them had academic education. Besides, on average, 17.31 ± 5.81 years had passed since they lived together (some demographic characteristics of the participants are listed in Table 2). It should be noted that no significant statistical difference was observed between the two active control and intervention groups in terms of education, spuose’s education, spuose’s occupation, and the percentage of cancer in first degree relatives.

**Table 2:** Demographic characteristics of the studied samples in two groups

Group	Active control		Intervention	
Variable	Abundance	Percentage	Abundance	Percentage
Education				
Under diploma	8	24.2	6	17.1
Diploma	17	51.6	14	40
Academic	8	24.2	15	42.9
Spouse’s education				
Under diploma	9	27.7	8	22.8
Diploma	13	39.3	11	31.4
Academic	11	33.3	16	45.8
Job				
Housewife	27	81.8	22	62.8
Employed	4	12.1	11	31.4
Retired	2	6.1	2	5.8
Spouse’s job				
Freelance	10	30.3	10	28.5
Employee	8	24.4	11	31.4
Manual worker	12	36.3	8	22.8
Retired	3	9	6	17.3
History of cancer in 1st degree relatives				
Positive	7	21.2	10	28.5
Negative	26	78.8	25	71.5
History of cancer in 2nd degree relatives				
Positive	9	27.2	9	25.7
Negative	24	72.8	26	74.3

Based on the results, the mean scores of the sexual quality of life before the intervention in the studied groups (intervention and active control) were not statistically different. The mean scores of the sexual quality of life before the intervention, immediately after the intervention, and 1 month after the intervention were 68.57 ± 18.63, 78.84 ± 12.7, and 79.60 ± 19.88, respectively, which, as displayed in Table 3 by Mann-Whitney test, are significantly different (*P* < 0.05).

**Table 3:** Comparison of the mean score of the sexual quality of life in women with a history of breast cancer before, after, and 1 month after the study in the intervention and active control group

Group	Intervention	Active control	*P*-Value*
Variable	Mean ± SD	Mean ± SD	
Before intervention	68.57 ± 18.63	64.45 ± 22.76	0.38
Immediately after the intervention	78.84 ± 12.7	67.2 ± 20.29	0.009
1 month after the intervention	79.6 ± 19.88	78.7 ± 20.76	0.008
*P*-Value**	0.007		

^*^Mann-Whitney u

^**^Tests of Between-Subjects Effects (GLM)

The results of comparing the mean scores of the sexual quality of life of women in three time points separately in each group using the Wilcoxon test suggested that there was a significant increase in the mean score of the sexual quality of life in the intervention group in the period immediately after the intervention and 1 month after the intervention compared to before intervention (Table 4).

**Table 4:** Intra-group comparison of the mean score of the sexual quality of life in women with a history of breast cancer before, after, and 1 month after the study in the intervention and the active control groups

**Group**	**Time**	**Mean ± SD**	P*
Intervention	Before intervention	68.6 ± 18.6	0.015
	Immediately after the intervention	78.8 ± 12.8	
	Before intervention	68.6 ± 18.6	0.012
	1 month after the intervention	79.1 ± 12.1	
	Immediately after the intervention	78.8 ± 12.8	0.94
	1 month after the intervention	79.1 ± 12.1	
Active control	Before intervention	64.5 ± 22.8	0.53
	Immediately after the intervention	67.3 ± 20.3	
	Before intervention	64.5 ± 22.8	0.33
	1 month after the intervention	69.3 ± 19.9	
	Immediately after the intervention	67.3 ± 20.3	0.62
	1 month after the intervention	69.3 ± 19.9	

^*^Wilcoxon Signed Ranks Test

## Discussion

The main goal of this study was to investigate the effectiveness of online counseling with a solution-focused approach in the sexual quality of life of women with a history of breast cancer whose treatment period had ended. The results showed that the mean score of the sexual quality of life of the intervention group was statistically significantly different before the intervention, immediately after, and 1 month after intervention, and the use of online counseling with a solution-focused approach led to improvement of sexual quality of life score. So that in the period immediately after the intervention and 1 month after that, the sexual quality of life score of none of the participants of this group was in the poor range and most of them were in the range of the favorable score.

The results of the active control group revealed that the mean score of the sexual quality of life in this group was not statistically different compared to before the intervention. Moreover, 1 month after the intervention, the number of participants who were in the range of the appropriate score and the mean score of the sexual quality of life was greater than before the intervention and immediately after the intervention. Although the difference in the mean score of the sexual quality of life immediately and 1 month after the study was relative and lacked statisitcal significance, it seems that providing solution-focused content in a longer period of time may improve the sexual quality of life in women with breast cancer. According to the results of the study, the mean score of the sexual quality of life before the intervention in both groups was in the average range and there was no statistically significant difference between them. After the intervention, the mean score of the sexual quality of life increased in both groups; nonetheless, the increase in the mean score was higher in the intervention group than in the active control group, and this difference was statistically significant. Furthermore, the mean score of the sexual quality of life increased 1 month after the intervention compared to before in both groups; the increase in the intervention group was more than the active control group and the difference was significant. To confirm the sexual health disorder of women with breast cancer, Brajkovic et al. [34] conducted a systematic review and showed that women with breast cancer have different degrees of disorder in their sexual quality of life, and the most important cause of this disorder is related to mastectomy and radiotherapy. They recommended psychological interventions to improve the quality of women’s sexual health. By explaining the experiences of the sexual life of women with breast cancer, Maleki et al. emphasized that those women saw their sexual life as over and described themselves in a severe sexual crisis. These scholars also recommended the provision of support structures, counseling, and effective interventions to promote the sexual health of women with breast cancer [35]. The results of our study also demonstrated that the sexual quality of life score of a significant part of the participants (51.6% in the active control group and 42.4% in the online counseling group) in both groups was within the range of weak and moderate before the intervention, which can indicate the serious involvement of this group of patients with sexual problems and disorders. All these studies disclose the necessity of sex counseling for this group of women. Bokaie et al. [36] in a study entitled: “The effectiveness of group problem-solving therapy on the sexual performance and satisfaction of women after mastectomy” showed that sexual performance and satisfaction after problem-solving solution counseling is improved among mastectomized women. Although the mean score of sexual function improved from 18.37∓8.35 to 22.95 ± 5.79 in the follow-up period in their study, and though this increase was statistically significant (*P* < 0.0001), it was not clinically above the cut-off point (28 for Iranian women) [36]. The similarity between that study and our study was the holding of eight 90-min sexual health counseling sessions and a 1-month follow-up period; the differences between that study and the present study are that their counseling sessions were in person or face-to-face and there was no control group in that study. The results of Liu et al.’s clinical trial, that investigated the effect of solution-focused treatment on cancer fatigue in women with breast cancer undergoing chemotherapy, showed that the level of cancer-induced fatigue during the course of chemotherapy following the use of solution-focused counseling decreased compared to before the intervention, or its increase has been less compared to the control group [37]. In this study, the mean score of fatigue caused by cancer in the intervention group decreased from 4.34 ± 0.83 before the intervention to 3.24 ± 0.74 after the intervention (*P* = 0.023). However, in the 12-week follow-up period, the mean score of fatigue caused by cancer increased to 4.26 ± 0.86 (*P* = 0.174). Nevertheless, this increase is smaller and the difference is significant, though, compared to the mean score of the control group in the follow-up period (5.94 ± 0.75)) (*P* = 0.023). The similarity between this study and our study lies in the counseling approach used and the population under investigation, and the difference is the use of a 3-month follow-up period and a 5-session intervention. Amini Nasab et al. also reported in their study that the level of depression and perceived stress had decreased following the use of a solution-focused approach in breast cancer patients [38]. In this study, the mean scores of depression and perceived stress in the intervention group decreased from 43.07 ± 5.09 and 35.27 ± 3.78 before the intervention to 29.6 ± 5.11 and 23.53 ± 4.91 after the intervention, respectively (*P* > 0.001). In this study, similar to our study, a mild decrease was observed in the intervention group in the sexual quality of life score in the follow-up period. A slight increase was observed in the mean score of depression and perceived stress in the follow-up period (31.6 ± 4.38 and 24.73 ± 5.28, respectively), which can indicate the necessity of continuing counseling programs in the care period after treatment of breast cancer patients. This study is similar to our study in terms of the population under investigation, the duration of the follow-up period, the counseling approach, and the number of sessions used. In their clinical trial, Zhang et al. investigated the effect of the solution-focused approach on psychological distress, depression, anxiety, hope, and somatization in young participants and adolescents with cancer [39]. The results of the analysis of variance (ANOVA) in this study showed that following the use of four solution-focused counseling sessions in the intervention group, the investigated indicators including psychological distress (*P* < 0.001), depression (*P* < 0.01), and anxiety (*P* < 0.001) showed a significant reduction. Besides, the hope index showed a significant increase in the period immediately after the intervention compared to the active control group (*P* < 0.001). In the 1-month follow-up period, in addition to the mentioned indicators, the somatization index also improved significantly compared to the control group. This study is similar to our study in terms of follow-up period and counseling approach. In Novella et al.’s study, a comparison was made between two methods of online counseling and face-to-face counseling to reduce students’ anxiety using a solution-focused approach [40]. The results of this study showed that the use of solution-focused approach, both online and face-to-face, had caused a significant decrease in the mean score of Beck’s Anxiety Inventory after intervention in two stages of immediately after the intervention and the follow-up period (*P* = 0.001). The comparison between the two groups did not indicate a significant difference between the mean anxiety score between them (*P* = 0.580). This finding can confirm the hypothesis of our study, that is, the effectiveness of online counseling. This study is similar to our study in terms of using a solution-focused approach and the method of using online intervention. The difference was that they provided online counseling in the form of an individualized conversation for each person in the form of audio and video communication, while in our study, online counseling was provided in a group and in a collective space. In relation to the results of the present study, Abdul Nabi et al., in a quasi-experimental study, investigated the effectiveness of psychoeducational intervention on the sexual quality of life life, body image, and self-confidence of women with breast cancer [41]. Their intervention included a psychological and educational care program that was held face-to-face in 14 sessions of 30–90 min. In this study, counseling intervention has significantly improved the sexual quality of life of the participants. The index and the population under investigation in this study are similar to our study. The inventory used to check the level of sexual quality of life, lack of follow-up period, and counseling approach are among the differences between this study and ours. In this regard, Triberti et al. conducted a systematic review to investigate the role of eHealth interventions on the quality of life of women with breast cancer, and the results of their study showed the effectiveness of interventions in the field of electronic health in managing the health of breast cancer patients and improving their quality of life, especially interventions that use psychological approaches in the context of electronic health to improve patients’ abilities (such as coping and acceptance). However, the use of this platform is still limited in many aspects of women’s health, and researchers emphasized the use of interventions based on the eHealth platform to improve the quality of life of women with breast cancer [41]. The results of our study and the majority of similar studies show that counseling with a solution-focused approach can be used in the treatment of a significant range of mental and psychological disorders in various patient populations. The special features of the solution-focused approach, such as emphasizing individual abilities, effectiveness with a small number of sessions, not paying attention to past shortcomings and looking to the future, have made it a practical method for treating many mental and psychological disorders. The innovation of our study was the use of a solution-focused approach to improve the sexual quality of life in women with breast cancer. Counseling online and through social messengers with a solution-focused approach has also received much less attention in studies. Given the effectiveness of the solution-focused approach, researchers suggest that more studies in this field be caried out with a larger number of samples, with a more detailed design of pamphlets with solution-focused content, a longer follow-up period, with a focus on investigation of the effectiveness on other aspects of the sexual health of women affected with breast cancer, and even intervention in the beginning stages of breast cancer treatment.

## Study limitations

The cultural requirements of the studied community (Iran) were among the limitations of this study, which made some participants shy away from participating in a study on sexual issues. On the other hand, talking about cancer and recalling the bitter memories of the treatment period was annoying for a significant number of them. To overcome this limitation, all participants were assured that in this research, due to the use of a solution-focused approach, the main focus was on the abilities and capacities of participants, and talking about past memories and problems related to cancer was avoided. Moreover, each participant was assigned a pseudonym so that their identity would remain unknown during the counseling sessions. Besides, due to the weak internet infrastructure in some areas, sometimes the internet of the participants was disconnected and caused problems in the online sessions.

## Conclusion

The use of a solution-focused approach online can improve the sexual quality of life of women with breast cancer and reduce the sexual problems caused by this disease and its treatment. Thus, given the numerous sexual problems of women with cancer and considering the increase in the survival rate of patients with the aim of improving diagnostic and therapeutic methods, improvement of the sexual quality of life of these women is of utmost significance as one of the most important aspects of participants’s lives. Hence, it is recommended to consider the use of this approach in counseling and treatment centers to improve the sexual quality of life of women with breast cancer.

## Data Availability

The datasets used and analyzed during the present study are available from the corresponding author on reasonable request.

## Abbreviations

SQL-F: Sexual Quality of Life-Female
SFBT: Solution Focused Brief Therapy
